# How do students study in STEM courses? Findings from a light-touch intervention and its relevance for underrepresented students

**DOI:** 10.1371/journal.pone.0200767

**Published:** 2018-07-31

**Authors:** Fernando Rodriguez, Mariela J. Rivas, Lani H. Matsumura, Mark Warschauer, Brian K. Sato

**Affiliations:** 1 School of Education, University of California Irvine, Irvine, California, United States of America; 2 Department of Molecular Biology and Biochemistry, University of California Irvine, Irvine, California, United States of America; Charles P. Darby Children's Research Institute, 173 Ashley Avenue, Charleston, SC 29425, UNITED STATES

## Abstract

With the nationwide emphasis on improving outcomes for STEM undergraduates, it is important that we not only focus on modifying classroom instruction, but also provide students with the tools to maximize their independent learning time. There has been considerable work in laboratory settings examining two beneficial practices for enhancing learning: spacing and self-testing. In the current study, we examine biology students’ study practices, particularly in the context of these two behaviors. We specifically investigate whether a light-touch study skills intervention focused on encouraging spacing and self-testing practices impacted their utilization. Based on pre- and post-course surveys, we found that students report utilizing both beneficial and ineffective study practices and confirm that usage of spacing and self-testing correlates with a higher course grade. We also found that students in the section of the course which received the study skills intervention were more likely to report continued use or adoption of spacing and self-testing compared to students in control sections without the intervention. Surprisingly, we found that underrepresented minorities (URMs) under-utilize self-testing, and that our intervention helped to partially ameliorate this gap. Additionally, we found that URMs who reported self-testing earned similar course grades compared to non-URMs who also self-tested, but that there was a much larger drop in performance for URMs who did not self-test relative to non-URMs who also did not self-test. Overall, we would encourage instructors to dedicate class time towards discussing the merits of beneficial study practices, especially for students that have historically underperformed in STEM disciplines.

## Introduction

A number of reports have called for changes to existing educational practices to increase the quality and number of science, technology, engineering, and mathematics (STEM) graduates [1, 2]. This has produced many practices that fall under the loosely defined umbrella of active learning [1, 3, 4], which requires students to synthesize information or solve problems, often in collaboration with their peers. This differs from traditional lecturing, in which the role of the instructor is primarily confined to content delivery; instead, in active learning instructors are viewed as facilitators and students are required to take more responsibility for their learning by participating in a variety of activities designed to structure this learning [5, 6].

The need to improve STEM education is particularly salient in the context of the comparatively lower success rates for underrepresented minorities (URMs) in STEM programs [7]. A nationwide survey of students who entered college in 2004 found that 43% of White and 52% of Asian students who aspired to be STEM majors successfully completed a STEM degree within six years [8]. These figures were significantly lower for Latino/a (21.8%), African-American (24.9%) and Native-American (24.9%) students.

Considerable work has attempted to identify causes for the disparate success rates between URM and non-URM students. URM students tend to have access to lower quality STEM programs in their primary and secondary education [9], come from families and communities with a decreased understanding of the education system and potential career opportunities [10], and feel less motivation to pursue STEM degrees and decreased belongingness in STEM programs [11]. They can also have difficulty managing the academic environment in STEM higher education programs, a component of which is the development of effective study skills [12]. Thus, there is a need to utilize a wide variety of interventions to decrease the achievement gap between URM and non-URM students in STEM programs. While many current efforts emphasize how to best teach and deliver course content [3, 4], it is also vital that we improve how students spend their independent time outside of the classroom. This is particularly relevant for URM students who may be less familiar with means to self-regulate their learning [13].

### Spacing

There has been substantial work in laboratory settings examining various aspects of self-regulated study, the process by which individuals make decisions regarding what steps they take when studying on their own [14–16]. One such technique is spacing, where an individual’s study time is separated into multiple successive sessions as opposed to a much smaller number of lengthier study sessions [17]. A key aspect of spacing is that the same information is being incorporated across the study sessions, providing learners with multiple opportunities to review the material. This has been shown to be effective for long-term learning in a variety of different scenarios, from acquiring and maintaining vocabulary from a foreign language [18] or remembering words on a list [19], among others [20]. In an example of a typical spacing experiment, students were exposed to a mathematical tutorial, with a control group receiving ten practice questions in one sitting and the spacing group receiving five questions in each of two study periods. The spacing group significantly outperformed the control group on a follow-up test weeks later [21]. Spacing is proposed to be beneficial because learners tend to forget information between periods of learning (i.e. between two study sessions), thus allowing the learner to leverage the later sessions as opportunities to refresh oneself with a particular topic [22].

### Self-testing

Another beneficial study practice is self-testing, which involves assessing one’s understanding by solving practice problems. This learning strategy gives students opportunities to identify material they do and do not know, outside of a high-stakes testing environment. Additionally, it allows for retrieval practice, where repeatedly retrieving information from memory aids in easier retrieval later on, for example during an exam [23]. Multiple reviews emphasize that testing is one of the most well-established techniques known to aid in student learning [16, 20, 24], yet it has also been demonstrated that students under-utilize it in practice [14]. An example of the testing effect was shown by Butler and Roediger [25], where study participants were presented with art history lectures, and received either no additional material, lecture summaries, or short answer practice questions. Students who were provided with the practice questions outperformed the other groups when taking an exam one month later.

In light of the spacing effect, one question has been whether benefits of self-testing are due primarily to re-exposure to the tested material. Roediger and Karpicke explored this question using an experiment where participants were exposed to passages about scientific topics and either given an opportunity to re-study the material or provided with tests covering the material found in the passages [26]. Those who had the opportunity to re-study the material outperformed the testing group on an immediately administered test, but those in the self-testing condition performed better on a test given a week later. Additional work has shown that when both practices are presented, an additive impact is observed, as the combination of practice tests and repeated, spaced exposure to the material produces higher learning gains than either practice used alone [27].

The benefits of spacing and self-testing have been observed in K-12 classrooms as well as in higher education [24, 28–30], although both to a much smaller degree relative to the positive results that have been observed in laboratory settings. The potential impact of these practices in a college-setting is very intriguing, especially in light of the need to improve the quality of education with low-cost interventions. Not surprisingly, there have been multiple publications speculating as to how to best turn theory into practice for undergraduates [16, 17, 20, 24], for example, by structuring a course in a specific fashion. In a study by Hopkins et al., students were assigned required practice problems throughout the course, which mimicked the spacing effect, leading to greater student learning [29]. In the case of self-testing, Lyle and Crawford conducted an experiment where the course instructor devoted the last ten minutes of each lecture for students to answer practice questions regarding that day’s material [30]. The study found that students who were presented with the ungraded practice questions scored significantly higher on class exams relative to students who did not receive them. Ideally though, the use of particular study skills would be a conscious decision made by students, allowing them to apply these techniques to all of their courses independent of how the instructor structures the class, as well as in other aspects of their daily lives.

One barrier to implementation is that knowledge regarding the value of these practices may not be sufficient. Susser and McCabe found that while students recognized spacing was more beneficial than cramming, they were not more likely to utilize it relative to other, less effective strategies [31]. Kornell and Bjork surveyed a group of undergraduate students in an introductory psychology course, asking whether they studied based on what their teachers taught them about studying. 80% of respondents answered “No” to this question [14]. Similar results were observed by Morehead et al., despite the fact that nearly 80% of instructors claimed to discuss study techniques in their courses [32].

In this study, we examine the effectiveness of a light-touch study skills intervention with the goal to impact study practices utilized by students in a large enrollment molecular biology course. Rather than alter the course structure to force a certain behavior, we aim to see whether having an instructor repeatedly recommend spacing and self-testing as effective study strategies can alter how students study. This work investigates the following research questions:

1. How do students in a large enrollment STEM course study, and to what extent do students report utilizing spacing and self-testing strategies?
2. Does using spacing and self-testing strategies vary based on URM status?
3. Does a study skills intervention focused on spacing and self-testing impact student use of these strategies?
4. Does self-reported utilization of spacing and self-testing correlate with course performance?

Overall, we found that students in this biology course used a variety of study strategies, including those that have been identified as beneficial practices such as spacing and self-testing. Surprisingly, we found that URMs were less likely to implement self-testing, which may contribute to the performance gap these students exhibit. Our intervention resulted in higher self-reported usage of spacing and self-testing and closed the self-testing gap for URMs in one of the study years. We also found that student self-reporting of spacing or self-testing correlated with higher course performance, particularly for those who maintained use of each practice over the entire quarter, as opposed to those who had newly adopted the practice during the quarter. This implies that, in order for spacing and self-testing to be beneficial for learning, they need to be consistently practiced over a longer period of time, as opposed to a single quarter. This may be particularly true for at-risk students who may be less familiar with techniques that will help to self-regulate learning.

## Materials and methods

### Study setting

This study was performed at a large, PhD-granting research institution in the Western United States. Participants were students enrolled in one of three sections of a 10-week long molecular biology course. We studied these three course sections in two different years—Spring 2016 (Year 1) and Spring 2017 (Year 2). For both years, students in the intervention section were taught by one of the study authors (BKS), while students in the two control sections (C1 and C2) were taught by two distinct pairs of instructors. All sections were taught similarly with common lecture slides, periods of active learning during the lecture period, and identical, problem-based discussion sections. One distinction was that the intervention section had three midterm exams and one cumulative final versus the control section which had a single midterm exam and a cumulative final.

### Participants

Our sample for this study consisted of molecular biology course students who completed both pre- and post-surveys. For Year 1 of the study, 544 of the 937 enrolled students (58.2%) completed both pre- and post-surveys. In Year 2, 782 of the 1076 enrolled students (72.7%) completed both pre- and post-surveys. The majority of students were second year Biological Sciences majors, with smaller numbers of Pharmaceutical Sciences and Public Health majors. Additional information on student demographics can be seen in Table 1.

**Table 1:** Demographic profile of survey respondents in Year 1 and 2.

	Year 1 Respondents (N = 544)		Year 2 Respondents (N = 782)	
	N	Mean (SD) / Percentage	N	Mean (SD) / Percentage
Age	544	20.67 (1.94)	782	20.76 (2.06)
Female	338/543	62.13%	503/777	64.73%
Male	205/543	37.75%	274/777	35.26%
Asian	316/544	58.08%	402/782	51.40%
White	105/544	19.30%	148/782	18.92%
Hispanic/Latino	66/544	12.13%	145/782	18.54%
International	32/544	5.88%	47/782	6.01%
Other/Unknown	25/544	4.59%	40/782	5.11%
URM^1^	80/544	14.70%	169/782	21.61%
First Generation	236/525	44.95%	372/759	49.01%
Low Income	176/544	32.35%	272/781	34.82%
1st Year	98/544	18.01%	168/782	21.48%
2nd Year	388/544	71.32%	508/782	64.96%
3rd Year	46/544	8.45%	92/782	11.76%
4th Year	11/544	2.02%	10/782	1.27%
5+ Years	1/544	0.00%	4/782	0.51%
SAT Total Score	463	1808.51 (205.52)	658	1789.80 (212.49)
High School GPA	433	4.06 (0.19)	600	4.06 (0.19)
Cumulative College GPA	544	3.28 (0.44)	782	3.20 (0.48)
Final Course Grade	544	7.99 (2.83)	782	8.21 (2.73)

Demographic data for students who completed both the pre- and post-surveys are listed above.

^1^Underrepresented minorities (URMs) were categorized as Hispanic/Latino, African American, and Native American. Descriptive information for African American and Native American students are not reported individually due to small sample sizes and are categorized as Other/Unknown in this table. Final Course Grade ranged from 1 (F) to 13 (A+).

### Procedure

Students completed a study skills survey presented online at the beginning of week 1 (pre-survey) and the end of week 10 and finals week (post-survey) of the quarter. Survey completion was voluntary, and students were awarded one point of extra credit for taking each. The pre-survey asked students about their general study patterns (spacing vs. cramming) and specific study strategies (self-testing and other strategies).

During the second week of class, after the pre-survey closed, students in the intervention section were given a ten-minute lecture regarding beneficial study practices. The focus of these slides was on the benefits of spacing one’s studying and self-testing. Additionally, the instructor reminded students every week of the quarter that they should be using these behaviors. Instructors in the control sections did not discuss the recommended study habits.

The post-survey asked students to reflect on what study strategies they utilized in the course during the quarter. The slides that were used for the intervention can be found in S1 File.

### Survey instruments

We used a slight modification of Morehead and colleagues [32] measures to assess students’ study skills. This questionnaire asked students to self-report one, their general study patterns (spacing vs. cramming), and two, specific study strategies (self-testing, re-reading, condensing notes, cramming, etc.).

There were two questions that we used to create a spacing versus cramming category. Students were categorized as utilizing spacing if one, they answered “*I most often space out my study sessions over multiple days/weeks*” to the study patterns question, and two, they did not select “*absorb lots of information right before the test*” in the study strategies question. Students were categorized as utilizing self-testing if they selected “test yourself with questions or practice problems” as one of their top three study strategies.

When analyzing students’ spacing and self-testing strategies, we combined students’ pre- and post-survey responses to create four categories that captured pre to post patterns of how students utilized these study strategies (1 = used strategy, 0 = did not use strategy). For both strategies (spacing, self-testing), students were coded as (1) never used strategy (0 = pre, 0 = post), (2) stopped using strategy (1 = pre, 0 = post), (3) started using strategy (0 = pre, 1 = post), and (4) maintained using strategy (1 = pre, 1 = post).

In order to understand how students strategize their studying, we asked the following open-ended question: “When studying, how do you generally decide what class to study for first?” S2 File contains the questions from the pre- and post-surveys.

### Learning management system clickstream data

For Year 1 of the intervention section, we obtained students’ overall mouse click activity in the learning management system (LMS). The LMS was used by the instructor to post assignments, pre-lecture quizzes (required prior to every course lecture), lecture slides, homework assignments (due weekly) and links to lecture podcasts, among other items, and housed the course discussion board. Because of the wide variety of resources and assignments contained within the LMS, we feel that overall student click activity provides an overview of out-of-class student engagement with the course.

### Institutional demographic and achievement data

Additionally, student demographic data (age, gender, ethnicity, low-income, and first-generation status) and prior achievement data (SAT total score) were obtained from the school registrar. At least one piece of these data were missing for 15.1% of students in Year 1 and 16.3% of students for Year 2. These students were removed from the analyses. The overall final grade from each course was used as outcome data. This score ranged from 1 (grade F) to 13 (grade A+). All study activities were performed under the approval of the University of California Irvine Institutional Review Board (HS# 2015–2225).

### Data analysis strategy

When assessing students’ survey responses at week 1, we used chi-square analysis to compare group differences (URM vs. non-URM, intervention vs. control section) in students’ utilization of specific study strategies (1 = used strategy, 0 = did not use strategy). Chi-square was also used to examine group differences in students’ study patterns at pre and post. T-tests were used to compare the overall relationship between students’ study strategies at week 10 and their final course grade. ANOVA was used to compare differences between URM and non-URMs across different course sections. Finally, we used regression analysis to predict students final course grades while controlling for course section, demographics, and prior achievement.

Survey responses to the question “When studying, how do you generally decide what class to study for first?” were coded using an iterative, inductive process [33]. Two members of the study team independently read through the survey responses to develop the coding scheme. Using this scheme, they coded the first 15% of survey responses. Because students’ responses were very straightforward, the two team members achieved 99.84% agreement. Due to the high level of accuracy between the coders, one member coded the remaining responses alone.

Course LMS data was analyzed by isolating individual student’s click activities over the course of the quarter. Aggregating student’s average clicks per day over the ten weeks of the course allowed us to examine whether self-reported spacing versus cramming was reflected in the students’ engagement with the LMS. To particularly identify student behavior in the context of exams, we focused on student activity in the five days prior to each course midterm.

The minimal data set is publicly available and can be found here: https://dataverse.harvard.edu/dataverse/2018studyskills

## Results

### Self-reported study behaviors at week 1

**Fig 1:**
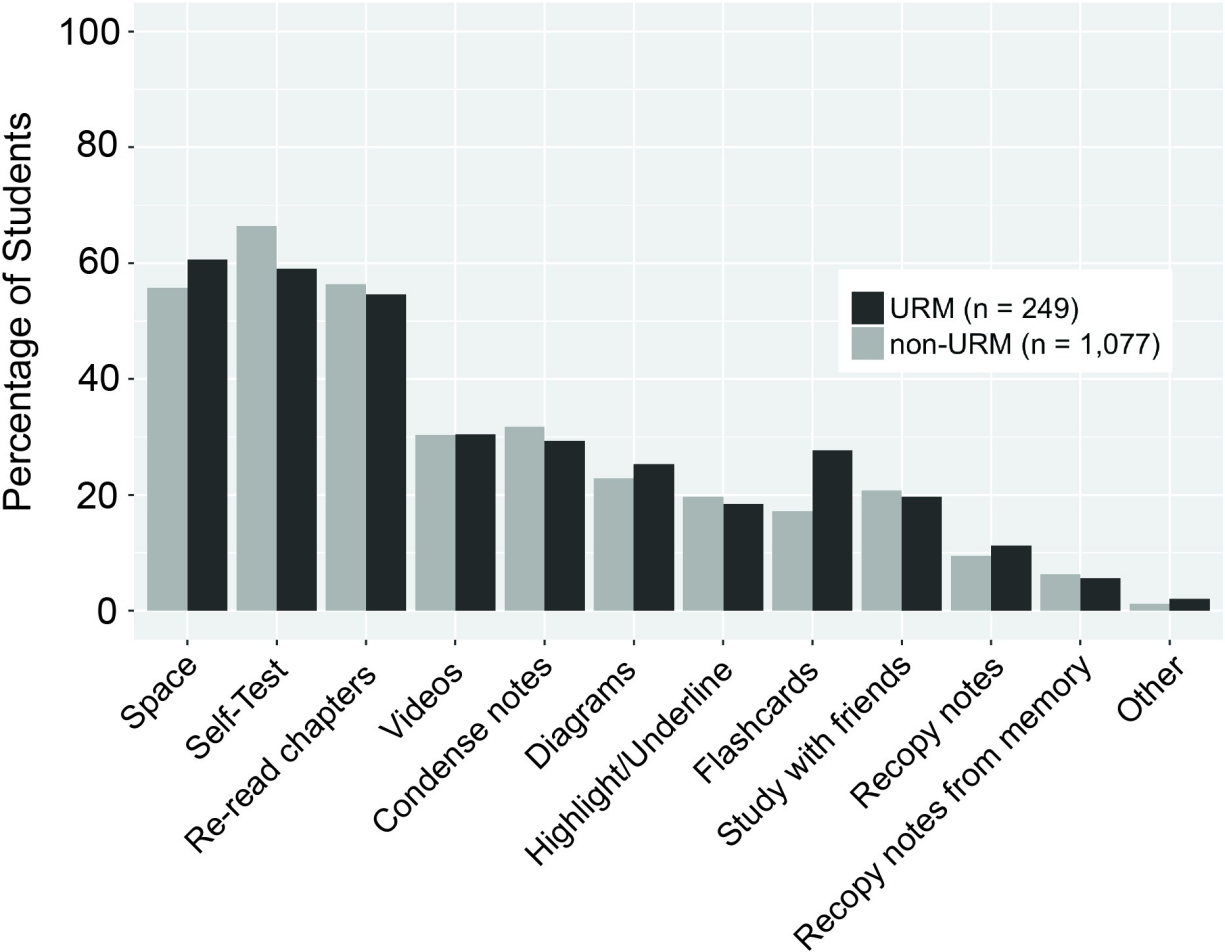
Underrepresented minority (URM) and non-URM students’ self-reported study patterns. Students enrolled in the study course sections reported their study patterns in a pre-course survey from years 1 and 2 of the study. The spacing designation was determined if (1) students answered “I most often space out my study sessions over multiple days/weeks” to the question “Which of the following best describes your study patterns?” and (2) they did not select “absorbing lots of information the night of the test” responding to the question “Select the top 3 study strategies you use most regularly.” The study strategies were determined from response to the latter question. Chi-squared tests determined whether the fraction of URM and non-URM students who selected each strategy were significantly different. *p < .*05*, **p < .*01*.

Students were asked to report in the pre-course survey whether they space their studying and to indicate the top three study strategies they regularly use. When looking at the overall study patterns across Years 1 and 2, prior to any intervention, 56.7% of students reported spacing their studying versus 43.3% who reported cramming. There was a wide range of study strategies reported with the most common being self-testing and re-reading a textbook (Table 2). Other commonly cited strategies included watching videos, condensing notes, making diagrams, and highlighting. We examined whether study practices varied with URM status and found that URM students reported utilizing self-testing to a significantly lower degree (59.0% for URMs compared to 66.4% for non-URMs) [*X*^*2*^ (1, *N =* 1,326) = 4.48, *p* < .05]. URMs also reported using flashcards to a higher degree than their non-URM peers (27.7% compared to 17.2%) [*X*^*2*^ (1, *N =* 1,326) = 13.81, *p* < .01] (Fig 1).

**Table 2:** Percent of Self-Reported Study Strategies (pre-survey).

		Overall (N = 1,326)
Study Patterns		
	Spacing	56.71%
Study Strategies		
	Self-Testing	65.00%
	Re-Read Chapters	56.03%
	Watch Video Lectures	30.31%
	Condense Notes	31.29%
	Make Diagrams	23.30%
	Study with Friends	20.58%
	Highlight/Underline Text	19.45%
	Flashcards	19.15%
	Recopy Notes	9.80%
	Recopy Notes from Memory	6.18%
	Other	1.35%

Student responses to the questions identifying whether students space their studying and the most common strategies they utilize. The spacing designation was determined if (1) students answered “I most often space out my study sessions over multiple days/weeks” to the question “Which of the following best describes your study patterns?” and (2) they did not select “absorbing lots of information the night of the test” responding to the question “Select the top 3 study strategies you use most regularly.” The study strategies were determined from response to the latter question.

We also examined how students decided which of their courses they chose to study for at a given time (Table 3). Students commonly cited that they prioritized studying the for courses with the most challenging material, followed by courses with the soonest upcoming quizzes or assignments. These responses are similar to those identified in previous work [27]. URMs did not show distinct responses for these strategies. For instance, 47.8% of URM students indicated that they prioritized studying for courses with the most challenging material compared to 44.1% of non-URMs [*X*^*2*^ (1, *N =* 1,224) = 0.86, *p* = .35]. URMs and non-URMs also had equal proportions for stating they studied whatever was due soonest (39.73 and 44.41%, respectively) [*X*^*2*^ (1, *N =* 1,224) = 1.46, *p* = .22].

**Table 3:** Percent of students’ study decisions (pre-survey).

	Overall (N = 1,224)	URM (n = 230)	Non-URM (n = 994)
Most challenging	44.85%	47.82%	44.16%
Whatever is due soonest	43.53%	39.73%	44.41%
Most Important	8.41%	9.56%	8.14%
Scheduling	8.57%	10.43%	8.14%
Easiest	4.41%	3.04%	4.72%
Most Interesting	3.35%	3.47%	3.32%
Other	7.11%	5.21%	7.55%

Student responses to the question “When studying, how do you generally decide what class to study for first?” were coded using an iterative process described in the methods. Responses were also broken down for respondents who were classified as underrepresented minorities (URMs) and non-URMs.

### Impact of the study skills intervention on spacing and self-testing

**Fig 2:**
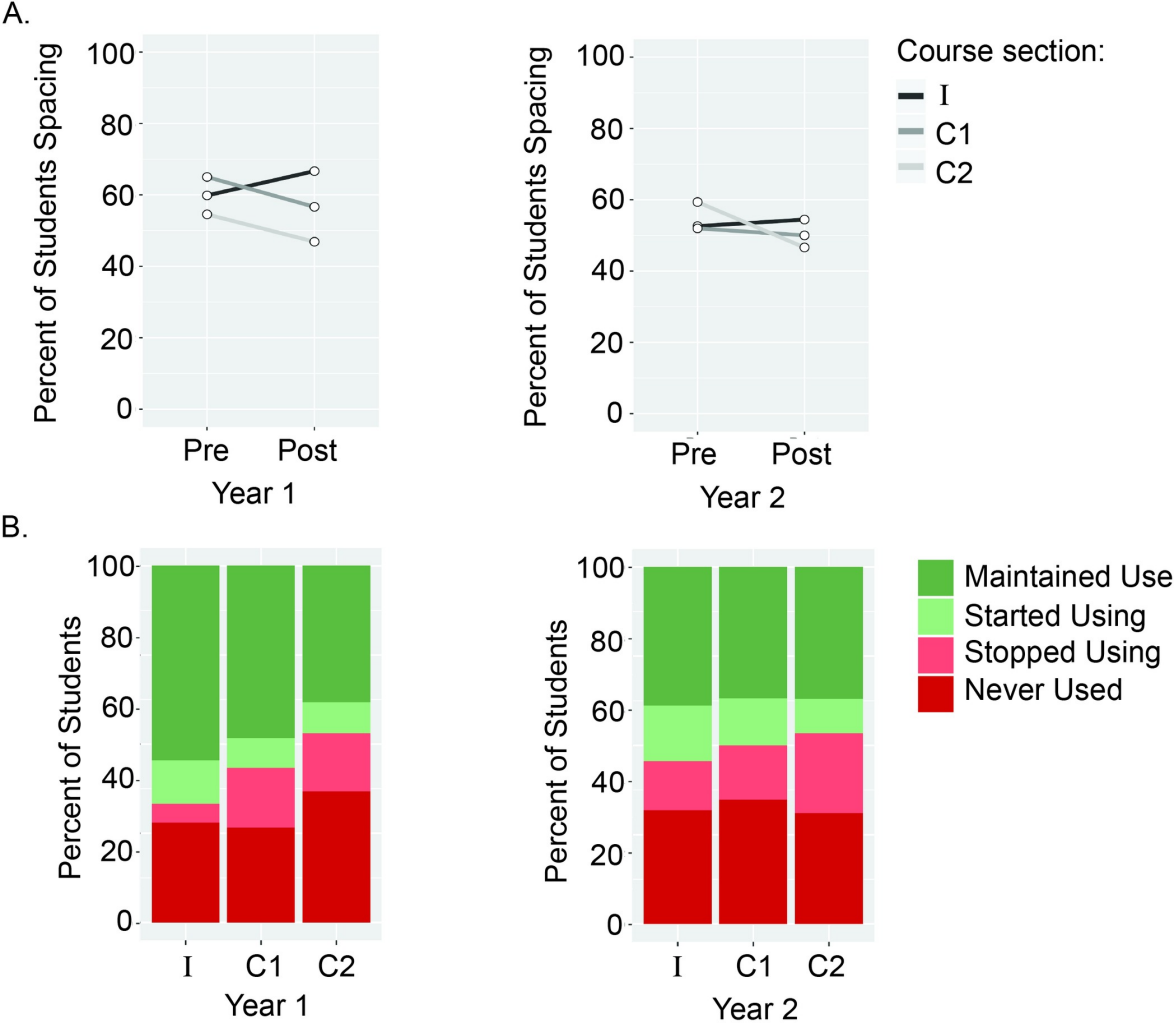
Impact of a study skills intervention on students’ self-reported spacing of their study. **(A).** Student responses to the spacing question on the pre- and post-course survey are reported for the control (C1 and C2) and intervention (I) sections during years 1 and 2 of the study. **(B).** Student spacing patterns are reported from the pre- and post-course surveys during years 1 and 2 of the study. *Maintained use* means the student reported spacing on both the pre- and post-survey, *started using* means the student only reported spacing on the post, *stopped using* means the student only reported spacing on the pre, and *never used* means the student did not report spacing on either the pre- or post-survey.

The percent of students stating that they spaced out their studying at week 1 (pre-survey) was similar across sections [*X*^*2*^ (2, *N =* 544) = 4.70, *p* = .10, for Year 1; *X*^*2*^ (2, *N =* 782) = 3.39, *p* = .18, for Year 2]. By week 10 (post-survey), there were significant differences in self-reported spacing by section for Year 1 [*X*^*2*^ (2, *N =* 544) = 13.03, *p* < .01] but not for Year 2 [*X*^*2*^ (2, *N =* 782) = 3.54, *p* = .17]. When examining the basic percentage changes of students using spacing between the pre- and post-surveys, in Year 1, the intervention section had a 6.8% *increase* while control sections C1 and C2 had an 8.4% and 7.7% *decrease*, respectively. In Year 2, the intervention section had a 1.8% *increase* and control sections C1 and C2 had a 2.0% and 12.8% *decrease*, respectively (Fig 2A).

We then examined student spacing patterns over the course of the quarter in the context of whether they were enrolled in the intervention or control sections. These patterns included maintained use (reported on both the pre- and post-survey), started using (only reported on the post), stopped using (only reported using on the pre) and never used (did not report on either the pre- or post-survey). The differences in students spacing patterns over the quarter differed by section for Year 1 [*X*^*2*^ (6, *N =* 544) = 19.51, *p* < .001], although this effect was only marginally significant for Year 2 [*X*^*2*^ (6, *N =* 782) = 11.31, *p* = .07] (Fig 2B). For Year 1, we conducted post-hoc tests comparing differences in the proportions among the spacing patterns between the intervention and control sections (see Table 4 for percentages by course section and S1 and S2 Tables for the post-hoc results). For these post-hoc tests, we particularly focused on comparing whether the proportions between maintaining versus stopped using spacing were significantly different for the intervention section when compared against control sections. Post-hoc tests revealed that the intervention section had a higher proportion of students maintaining their spacing throughout the quarter when compared to the control sections, both of which had higher proportions of students who stopped using spacing.

**Table 4:** Spacing and self-testing patterns by course section and year.

		Year 1 (N = 544)			Year 2 (N = 782)		
		Intervention (n = 132)	Control Section 1 (n = 203)	Control Section 2 (n = 209)	Intervention (n = 327)	Control Section 1 (n = 204)	Control Section 2 (n = 251)
Spacing							
	Maintained Use	54.54%	48.27%	38.27%	38.83%	36.76%	37.05%
	Started Using	12.12%	8.37%	8.61%	15.59%	13.23%	9.56%
	Stopped Using	5.30%	16.74%	16.26%	13.76%	15.19%	22.31%
	Never Used	28.03%	26.60%	36.84%	31.80%	34.80%	31.07%
Self-Testing							
	Maintained Use	50.75%	32.01%	37.32%	52.29%	34.80%	31.07%
	Started Using	21.21%	8.37%	9.56%	14.37%	10.78%	9.96%
	Stopped Using	12.87%	28.07%	27.75%	15.90%	30.88%	33.86%
	Never Used	15.15%	31.52%	25.35%	17.43%	23.52%	25.09%

Students in either the intervention or one of the control course sections were classified based on their self-reported usage of spacing and self-testing. *Maintained use* means the student reported using that strategy on both the pre- and post-survey, *started using* means the student only reported using the strategy on the post, *stopped using* means the student only reported using the strategy on the pre, and *never used* means the student did not report using the strategy on either the pre- or post-survey.

While research that examines use of study strategies commonly collects self-reported data, one concern is whether they reflect actual behaviors. To triangulate our data, we obtained students’ click behaviors in the online course LMS for Year 1 of the intervention section. When viewing the clickstream data (student clicks per day in the LMS over time) in the context of whether they reported spacing or cramming at week 10. We focused specifically on the five days leading up to the course midterm exams and found that students who self-reported that they spaced were more likely to access the learning managements system earlier and more often prior to the midterm (S1 Fig). Both self-reported spacers and crammers had sharp increases in activity during the day of the midterm. While this does not completely validate student’s self-reported behaviors, it does present an independent data source to support behavioral differences in students who claim to space or cram.

**Fig 3:**
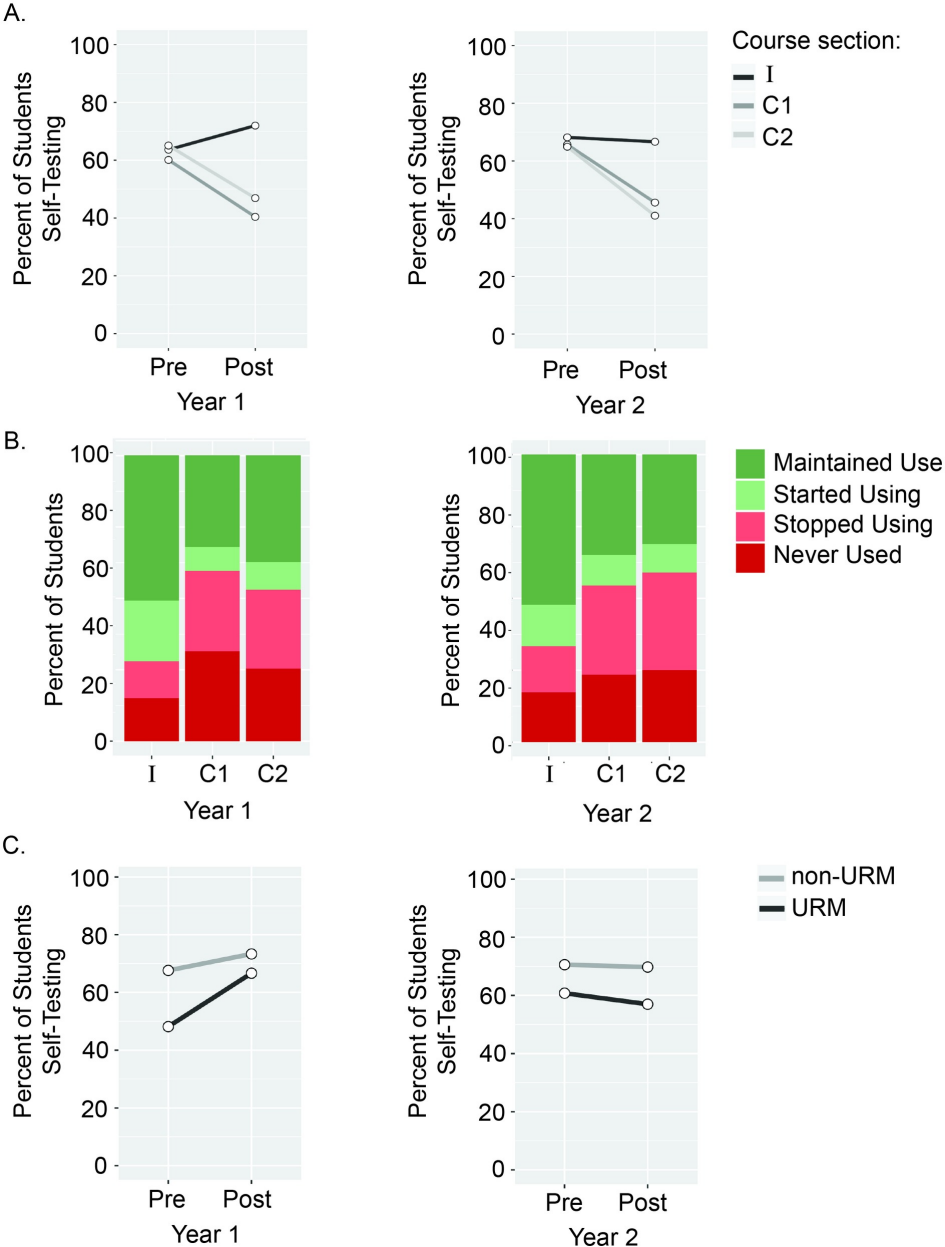
Impact of a study skills intervention on students’ self-reported self-testing. **(A).** We examined the percent of student responses that designated self-testing as one of their top three study strategies on the pre- and post-course survey are reported for the control (C1 and C2) and intervention (I) sections during years 1 and 2 of the study. **(B).** Student self-testing patterns were reported from the pre- and post-course surveys during years 1 and 2 of the study. *Maintained use* means the student reported self-testing on both the pre- and post-survey, *started using* means the student only reported self-testing on the post, *stopped using* means the student only reported self-testing on the pre, and *never used* means the student did not report self-testing on either the pre- or post-survey. **(C).** Students in the intervention sections during years 1 and 2 of the study were classified based on their URM status.

The percent of students reporting using self-testing was similar across the three sections at the beginning of the course [*X*^*2*^ (2, *N =* 544) = 1.13, *p* = .56, for Year 1; *X*^*2*^ (2, *N =* 782) = 0.75, *p* = .68 for Year 2]. By the post-survey, there were significant differences in self-reported self-testing by section [*X*^*2*^ (2, *N =* 544) = 33.72, *p* < .001 for Year 1; *X*^*2*^ (2, *N =* 782) = 43.43, *p* < .001 for Year 2]. When examining the basic change in the percentage of students using self-testing between the pre- and post-surveys, in Year 1, the intervention section had an 8.3% *increase* while control sections C1 and C2 had a 19.7% and 18.2% *decrease*, respectively. In Year 2, the intervention section had virtually no change (1.5% decrease) while control sections C1 and C2 had sharp *decreases* (20.1% and 23.8%, respectively) (Fig 3A).

The differences in students self-testing patterns over the quarter differed by section for both Years 1 and 2 [*X*^*2*^ (6, *N =* 544) = 37.90, *p* < .001 for Year 1; *X*^*2*^ (6, *N =* 782) = 46.18, *p* < .001 for Year 2] (Fig 3B). Post-hoc tests for both Year 1 and Year 2 revealed that the intervention section had a higher proportion of students who maintained self-testing throughout the quarter compared to both C1 and C2, which each had a higher proportion of students who stopped using self-testing.

We examined the impact of the intervention specifically on URM students, who reported using self-testing to a lesser degree than their non-URM peers at the beginning of the course. For Year 1, URM students exhibited increases in using self-testing between the pre- and post-surveys, resulting in similar levels of use as non-URM students [*X*^*2*^ (1, *N =* 132) = 0.20, *p* = .65]. In Year 2, URMs had a lower percentage of using self-testing than non-URMs in the post-survey [*X*^*2*^ (1, *N =* 327) = 3.85, *p* < .05]. However, it is important to note that the percentage of students using self-testing remained consistent between the pre- and the post-survey for both URM and non-URMs in the intervention section (Fig 3C). In contrast, URM students in the control sections exhibited similar drops in usage of spacing and self-testing by the end of the quarter as their non-URM counterparts (S2 Fig).

### Impact of the study skills intervention on re-reading the textbook, condensing notes, and using flashcards

One concern with our study design is that variables between the intervention and control courses, besides the intervention itself, may have impacted the improved utilization of spacing and self-testing. To determine whether the impact of the intervention on study strategies was specific, we also examined pre/post self-reporting of three other common strategies whose use impacted student performance, re-reading the textbook, using flashcards, and condensing notes. Self-reported use of these strategies at the beginning and end of the academic quarters were similar for both the intervention and control sections, illustrating that the impact of the study skills intervention was specific for spacing and self-testing. (S3 Fig).

### Overall relationship between spacing and self-testing on student performance

We next examined whether self-reporting either spacing or self-testing at the end of the academic quarter correlated with course performance. Students who reported spacing or self-testing earned significantly higher grades across both years of the study relative to those who reported cramming (Table 5). These grade increases were in the range of 5.7–6.2% for those using spacing and 4.6–6.5% for self-testing. We also examined the list of other study strategies students reported and their impact on course grade, and found that besides spacing and self-testing, condensing notes was positively associated with course performance across both years with grade increases in the range of 5.0–5.3%. Interestingly, using flashcards was negatively related with course performance across both years with grade decreases in the range of 5.9–6.0%.

**Table 5:** Final course grade by Self-Reported Study Strategies (post-survey).

		Year 1 (n = 544)				Year 2 (n = 782)			
		Used Strategy		Did not Use	*t*	Used Strategy		Did not Use	*t*
			Grade	Grade			Grade	Grade	
		%	M (SD)	M (SD)		%	M (SD)	M (SD)	
Study Patterns									
	Spacing	55.33%	8.32 (2.63)	7.58 (3.01)	2.99**	50.76%	8.60 (2.71)	7.80 (2.69)	4.16***
Study Strategies									
	Self-Testing	50.55%	8.29 (2.70)	7.69 (2.92)	2.46**	52.94%	8.61 (2.66)	7.76 (2.74)	4.40***
	Re-Read Chapters	66.91%	7.79 (2.84)	8.40 (2.76)	-2.37*	59.20%	8.14 (2.69)	8.30 (2.79)	-0.77
	Watch Video Lectures	28.30%	7.93 (2.85)	8.02 (2.82)	-0.31	31.32%	8.04 (2.75)	8.29 (2.72)	-1.17
	Condense Notes	38.41%	8.48 (2.67)	7.69 (2.88)	3.25**	39.64%	8.52 (2.70)	8.00 (2.73)	2.64**
	Make Diagrams	16.36%	8.51 (2.47)	7.89 (2.88)	2.10*	18.92%	8.81 (2.51)	8.07 (2.76)	3.15**
	Study with Friends	19.66%	8.17 (2.71)	7.95 (2.85)	0.76	25.95%	8.32 (2.58)	8.17 (2.78)	0.67
	Highlight/Underline Text	27.02%	7.74 (2.97)	8.09 (2.77)	-1.24	21.99%	7.78 (2.83)	8.33 (2.69)	-2.26*
	Flashcards	12.13%	7.09 (2.83)	8.12 (2.81)	-2.77**	7.03%	7.00 (2.83)	8.30 (2.70)	-3.30***
	Recopy Notes	9.00%	7.55 (3.05)	8.04 (2.80)	-1.10	13.42%	7.34 (2.79)	8.34 (2.70)	-3.44***
	Recopy Notes from Memory	2.88%	7.84 (3.02)	8.00 (2.82)	-0.30	5.75%	7.53 (3.18)	8.25 (2.70)	-1.48

Final course grade was examined in the context of the particular study strategies students stated they used on the post-survey. For each study strategy, *t*-tests were used to compare grade differences between students who reported utilizing the study strategy compared to students who did not report using the strategy. Course grades were converted to numerical values for this analysis ranging from A+ (13) to F (1).

* *p* < .05

**p < .01

***p < .001

We further wanted to understand whether self-reported self-testing at the end of the quarter was correlated with final course grades for URM students. Because the number of URM students in each course section was low in Year 1, which ranged from 25 to 27, we focus this sub-analysis on Year 2, which had a higher number of URM students. In Year 2, the number of URM students in the intervention and two control condition courses were 79, 41, and 49, respectively.

**Fig 4:**
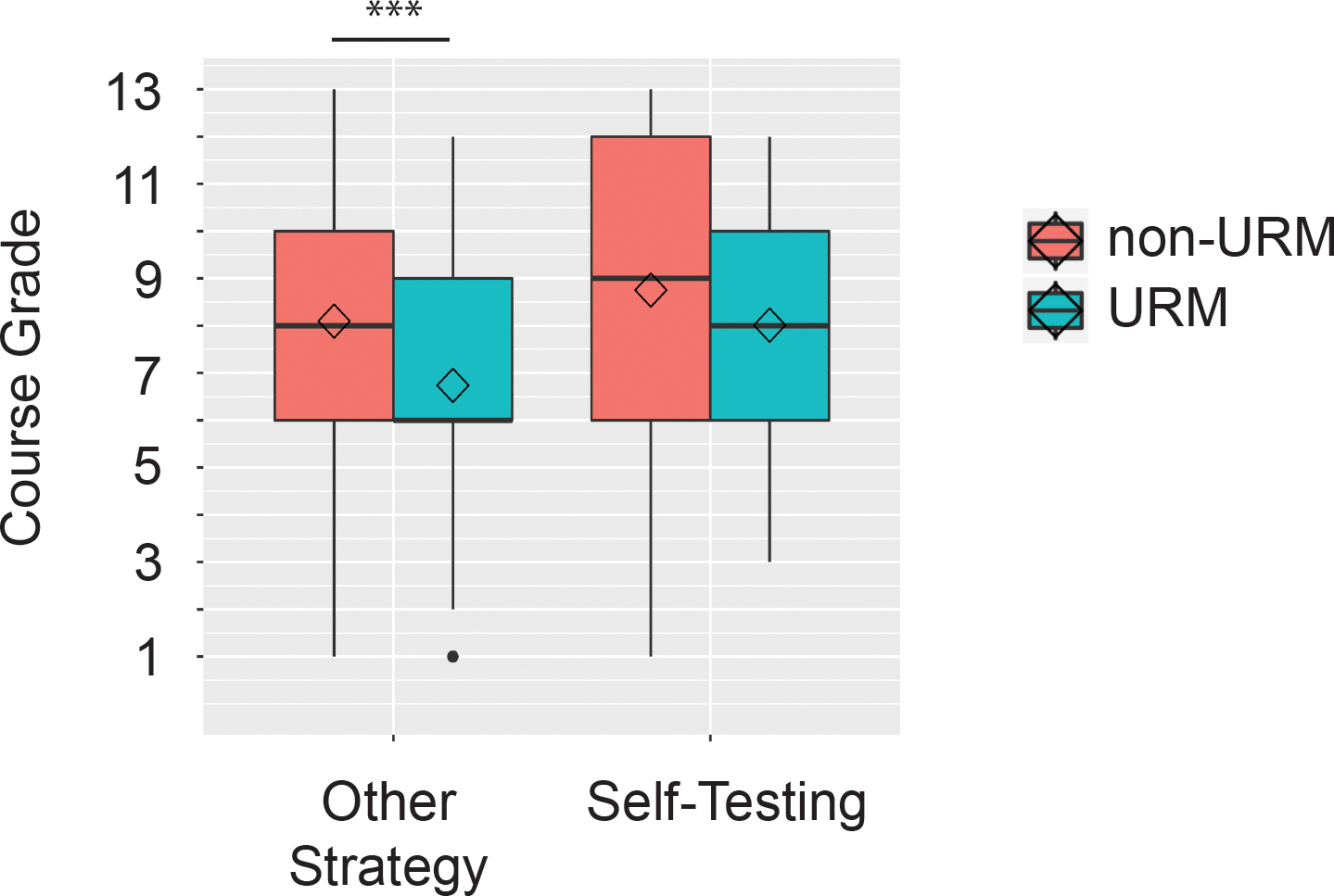
Course performance for URM and non-URM students based on used of self-testing. Course grades were examined for students who did and did not report self-testing based on their URM status. Grades were converted to numerical values for this analysis ranging from A+ (13) to F (1).

Using a 2 x 3 x 2 ANOVA (URM status, course section, self-testing) we found that, overall, URMs obtained lower final course grades (*M* = 7.33, *SD* = 2.68) when compared to non-URM students (*M* = 8.46, *SD* = 2.70)[*F*(1, 770) = 23.91, *p* < .001]. But when examining how URMs who reported self-testing faired compared to non-URMs who reported self-testing, post-hoc analyses found no differences in final course grades (*p* adjust = .10) (Fig 4). URMs who self-tested did just as well (*M* = 8.01, *SD* = 2.47) as their non-URM peers who reported self-testing (*M* = 8.75, *SD* = 2.70). In contrast, URMs who did not report self-testing had significantly lower final course grades (*M* = 6.74, *SD* = 2.74) than non-URM students who also did not self-test (*M* = 8.10, *SD* = 2.66)(*p* adjust < .001). There were no differences between URM status and course section on final course grades [*F*(2, 770) = 1.72, *p* = .17]. There was also no interaction between URM status, course section, and self-testing [*F*(2, 770) = 2.33, *p* = .72].

We then examined the impact of the particular spacing and self-testing patterns students reported in the pre- versus post-survey on course grade while controlling for course section, year in college, gender, URM status, and SAT total score (z-score). For both Year 1 and Year 2 regression results, we found that maintaining spacing throughout the quarter, compared to never using spacing, positively predicted final course grade (Table 6). For self-testing, maintaining this strategy was positively correlated with course grade in Year 2. Interestingly, adoption of spacing or self-testing (students who reported doing so only in the post-survey) did not correlate with an increased final grade.

**Table 6:** Final course grade regressed study strategy categories, course section, demographic, and prior achievement.

	Year 1 (N = 462)			Year 2 (N = 655)		
Variable	Coeff	Std. Error	*t*-value	Coeff	Std. Error	*t*-value
(Intercept)	8.93	0.62	14.36***	8.14	0.47	17.45***
Spacing—Maintained	1.72	0.28	6.14***	1.23	0.23	5.40***
Spacing—Increased	0.74	0.43	1.72+	-0.11	0.30	-0.36
Spacing—Decreased	1.39	0.37	3.71***	0.47	0.28	1.69+
Self-Testing—Maintained	0.02	0.31	0.055	1.12	0.26	4.37***
Self-Testing—Increased	0.39	0.41	0.934	0.59	0.34	1.75+
Self-Testing—Decreased	0.04	0.33	0.121	0.47	0.28	1.71+
Condense Notes (Post)	0.42	0.24	1.72+	0.35	0.20	1.79+
Flashcards (Post)	-0.65	0.36	-1.79+	-1.36	0.37	-3.68***
Control Section 1	-0.70	0.31	-2.27*	0.58	0.23	2.51*
Control Section 2	-0.44	0.31	-1.419	0.19	0.23	0.841
Years Enrolled	-0.90	0.23	-3.97*	-0.73	0.16	-4.43***
Gender	0.39	0.24	1.606	0.47	0.20	2.33*
URM Status	0.20	0.35	0.552	-0.21	0.24	-0.88
SAT Total Score (z-score)	1.12	0.12	9.06***	0.91	0.10	9.04***

Course grade was examined in the context of a variety of factors including study pattern in regard to spacing and self-testing, usage of note condensing as self-reported on the post survey, usage of flashcards as self-reported on the post survey, course section, year enrolled at the university, gender, and URM status. Grades were converted to numerical values for this analysis ranging from A+ (13) to F (1). Because not all institutional data was complete (gender, SAT Total Score), missing data was 15.08% for Year 1, and 16.25% for Year 2. For Spacing and Self-Testing categories, students who did not use these strategies were dummy coded 0. The Intervention Course was dummy coded as 0. Additional dummy codes include Gender (0 = Female, 1 = Male) and URM Status (0 = non-URM, 1 = URM). Year 1 (*F*(14, 447) = 11.75, *p* < .001, Adj R2 = .25), Year 2 (*F*(14, 640) = 16.66, *p* <. 001, Adj R2 = .25)

+ *p* < .10

* *p* < .05

**p < .01

***p < .001

## Discussion

While there have been efforts to increase student achievement by implementing active learning practices in lecture, another improvement strategy involves teaching students how to be good learners outside the classroom. Prior to exposure to our study skills intervention, we found that our sample population of second-year molecular biology students reported using a mix of effective and ineffective study strategies, and that many (56.7%) already employed spacing as well as self-testing (65.0%). Surprisingly, use of self-testing was lower for URMs relative to their non-URM peers. We know that URMs typically come to college less academically prepared and exhibit lower performance metrics [34], and our results may provide some insight as to how differences in their study habits may contribute to this. The lower use of a beneficial study practice like self-testing may be due to the fact that URM students are disproportionately first-generation college students [35], individuals who have less knowledge regarding the university system and what is required for success.

Our light-touch intervention, which included a brief lecture on study skills as well as weekly reminders from the instructor, resulted in greater self-reported use of spacing and self-testing at the end of the course. More specifically, the intervention was most effective in helping students maintain self-testing strategies over the quarter. That is, for the students who already stated that they utilized self-testing strategies at the beginning of the course, the intervention helped them continue using this strategy. We also found that the intervention positively impacted their use of this strategy, even closing the self-testing gap between URMs and non-URMs in Year 1.

In terms of the overall relationship between spacing one’s studying or self-testing and course performance, we found that using these effective strategies was associated with higher final course grades. This supports previous studies highlighting the benefits of spacing and self-testing [16, 20, 24, 26]. We also found that the performance gap between URM and non-URM students was no longer observed when specifically looking at URMs and non-URMs that reported self-testing. These results point to the need to provide URM students with guidance about how to best study for a course and the potential value of promoting self-testing strategies.

Surprisingly, we found that adoption of spacing and self-testing (students who did not self-report using these practices at the beginning of the course, but did at the end) was not associated with higher course grades. Research on self-regulated learning has found that students have difficulty changing their study behaviors. Dembo and Seli [36] present a theoretical framework for understanding the processes that occur as a student tries to change his/her learning strategies. They posit that students need adequate practice with the strategies in order to implement them successfully. Past research has demonstrated that even when students are enrolled in courses entirely focused on study and learning strategies, they have a difficult time implementing those strategies in their courses [37]. As our current study was a light-touch intervention and did not include extensive instruction on how to implement the strategies, students who did not have previous experience with the strategies may have had a trouble implementing them successfully in order to positively impact their grades in the course.

When examining other strategies in relation to student outcomes, we found that condensing notes was positively correlated with higher final course grades, which we also identified in previous work [38]. Interestingly, using flashcards was negatively related to final course grade. While flashcards can be a beneficial study technique when used properly as a self-testing medium, students many not understand how to use them effectively. Some work suggests that flashcards are optimal for learning when using them involves recalling the “answer” prior to flipping over the card rather than simply re-reading the front and back of the card [15]. Using flashcards properly also involves spacing one’s study, such that the same cards are used many times over the study period, with temporal space in between study of each card [39].

While these findings have intriguing implications regarding the value of incorporating study skills training into the classroom, particularly for URMs, we want to highlight limitations of the work. First, the study did not randomly assign students to particular sections. Second, we rely on student self-report data. While self-report data is common in the study skills literature [14, 40, 41], students may be inaccurately reporting their study practices, either due to social desirability bias or lack of self-insight. For the post-survey responses, since the instructor gave explicit recommendations on how he believes students should study, this may have led students to over-report their use of these skills in order to please the instructor. However, self-report data is used quite often in psychological studies and has shown to be accurate when the respondent understands the question and when they do not fear reprisal [42]. Studies have also shown that web-based surveys suffer from less social desirability bias than other administration modes [43]. The differences observed in LMS clickstream activity between students who self-reported spacing and cramming also provides some evidence that student self-reports were accurate.

Another limitation of the study is that the impact of the intervention cannot be separated from instructor and course format effects. While the material studied in the intervention and comparison courses was similar, including the use of common lecture slides, discussion section activities, and online homework assignments, the intervention course contained more midterm exams (3) versus the control sections (1). The added exams in particular may have forced students to space their study to a greater degree. Still, there have not been reported impacts of increased numbers of exams on usage of self-testing, and our intervention had the most consistent impact on the use of self-testing, as opposed to spacing. Additionally, students in the intervention and comparison courses reported similar usage of three other common study strategies that were not emphasized by the intervention, re-reading textbook chapters, using flashcards, and condensing notes, providing additional evidence that the intervention, and not the course, produced our results.

While studies identifying beneficial impacts of altered teaching strategies (active learning) and course structure are important contributions to improve learning outcomes for STEM majors, an underappreciated area is an effort to improve students’ self-regulated learning. As students go through challenging STEM curriculum, improving skills that can translate across all of their courses could improve their overall success and confidence in the college. In particular, our finding that maintenance, as opposed to new adoption, of these strategies, correlated with improved performance, hints to the value of long-term study behaviors on performance. Much like any skill, it may be that studying with particular methods requires practice in order to become effective at those strategies. Reinforcement of these evidence-based strategies in multiple courses or at multiple time points in a student’s undergraduate career has the potential to transform the learning experience, particularly for URM or first-generation students who are likely to be less aware, and correspondingly more in need, of their benefits.

## Supporting information

S1 Fig Examination of student LMS clickstream data prior to each course midterm in the context of self-reported spacing. The average number of student clicks on the course learning management system (LMS) were reported for students who did and did not report spacing for the five days prior to each of the three course midterms.
(TIF)

S2 Fig Control students’ self-reported self-testing in the context of URM status. Students in the control sections (C1 and C2) during years 1 and 2 of the study were classified based on their URM status.
(TIF)

S3 Fig Impact of a study skills intervention on students’ self-reported re-reading chapters, and use of flashcards. The fraction of student responses that designated either re-reading the textbook **(A),** using flashcards **(B)**, or condensing notes **(C)** as one of their top three study strategies on the pre- and post-course survey are reported in the control (C1 and C2) and intervention (I) sections during years 1 and 2 of the study. The study skills intervention did not discuss either of these three study strategies.
(TIF)

S1 File Study skills intervention slides. (PDF)

S2 File Pre- and post-survey study skills items. (PDF)

S1 Table Post-hoc chi-square analysis of spacing and self-testing categories and course section (Year 1). Post-hoc tests were used to examine proportion differences for the different spacing and self-testing categories between the intervention and control courses. *Maintained use* means the student reported using that strategy on both the pre- and post-survey, *started using* means the student only reported using the strategy on the post, *stopped using* means the student only reported using the strategy on the pre, and *never used* means the student did not report using the strategy on either the pre- or post-survey. Note: Ns for each section are the following: Intervention Course = 132, Control Course B = 203, Control Course C = 209.
(PDF)

S2 Table Post-hoc chi-square analysis of self-testing categories and course section (Year 2). Post-hoc tests were used to examine proportion differences for the different self-testing categories between the intervention and control courses. *Maintained use* means the student reported using that strategy on both the pre- and post-survey, *started using* means the student only reported using the strategy on the post, *stopped using* means the student only reported using the strategy on the pre, and *never used* means the student did not report using the strategy on either the pre- or post-survey. Note: Ns for each section are the following: Intervention Course = 327, Control Course B = 204, Control Course C = 251.
(PDF)
