# Supplementary figures and images for: How do students study in STEM courses? Findings from a light-touch intervention and its relevance for underrepresented students
Source: PLoS One. 2018 Jul 31;13(7):e0200767. doi: 10.1371/journal.pone.0200767 (PMC6067695; doi:10.1371/journal.pone.0200767)

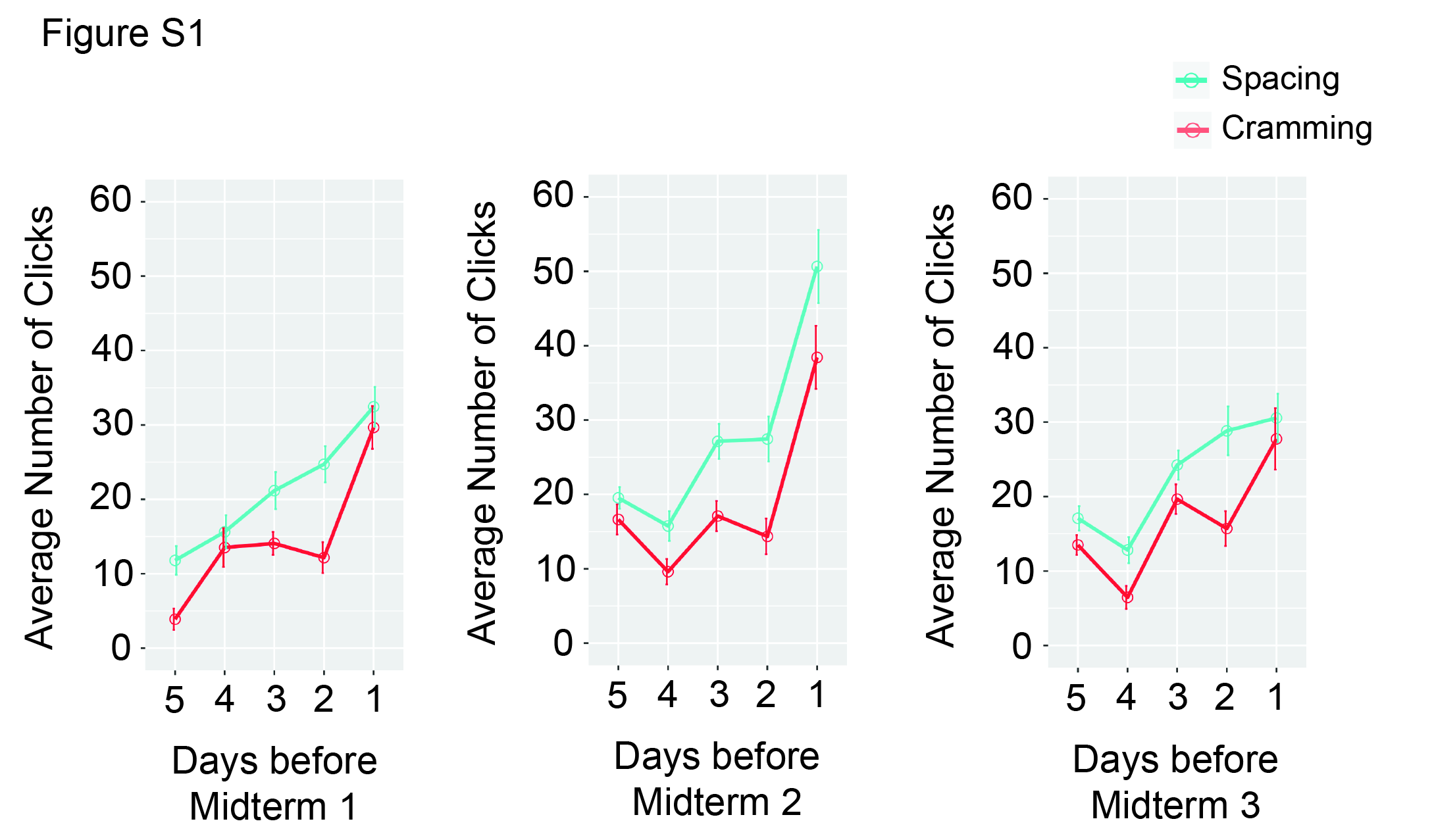

Supplement: S1 Fig — The average number of student clicks on the course learning management system (LMS) were reported for students who did and did not report spacing for the five days prior to each of the three course midterms. (TIF) [file pone.0200767.s001.tif]

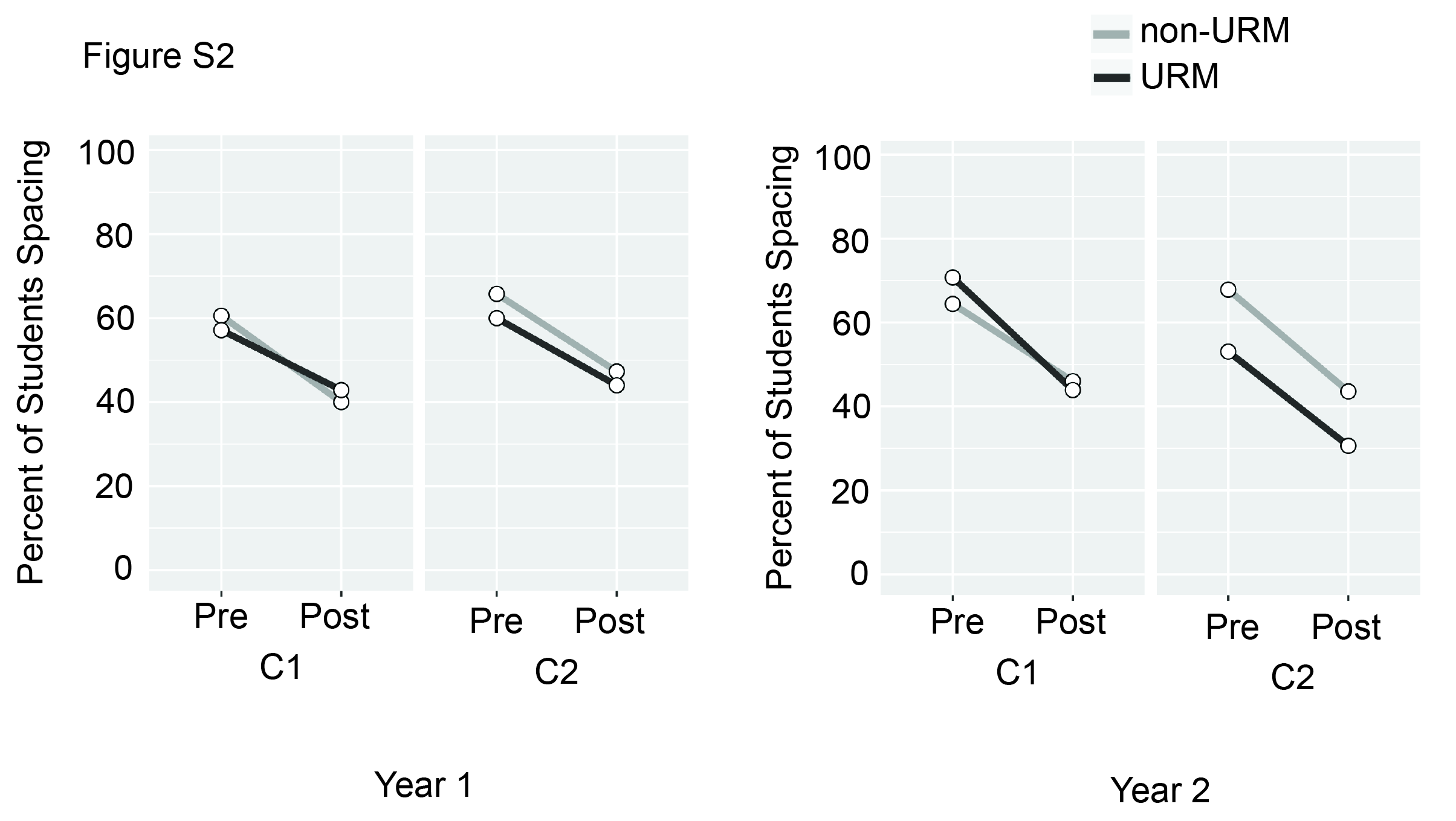

Supplement: S2 Fig — Students in the control sections (C1 and C2) during years 1 and 2 of the study were classified based on their URM status. (TIF) [file pone.0200767.s002.tif]

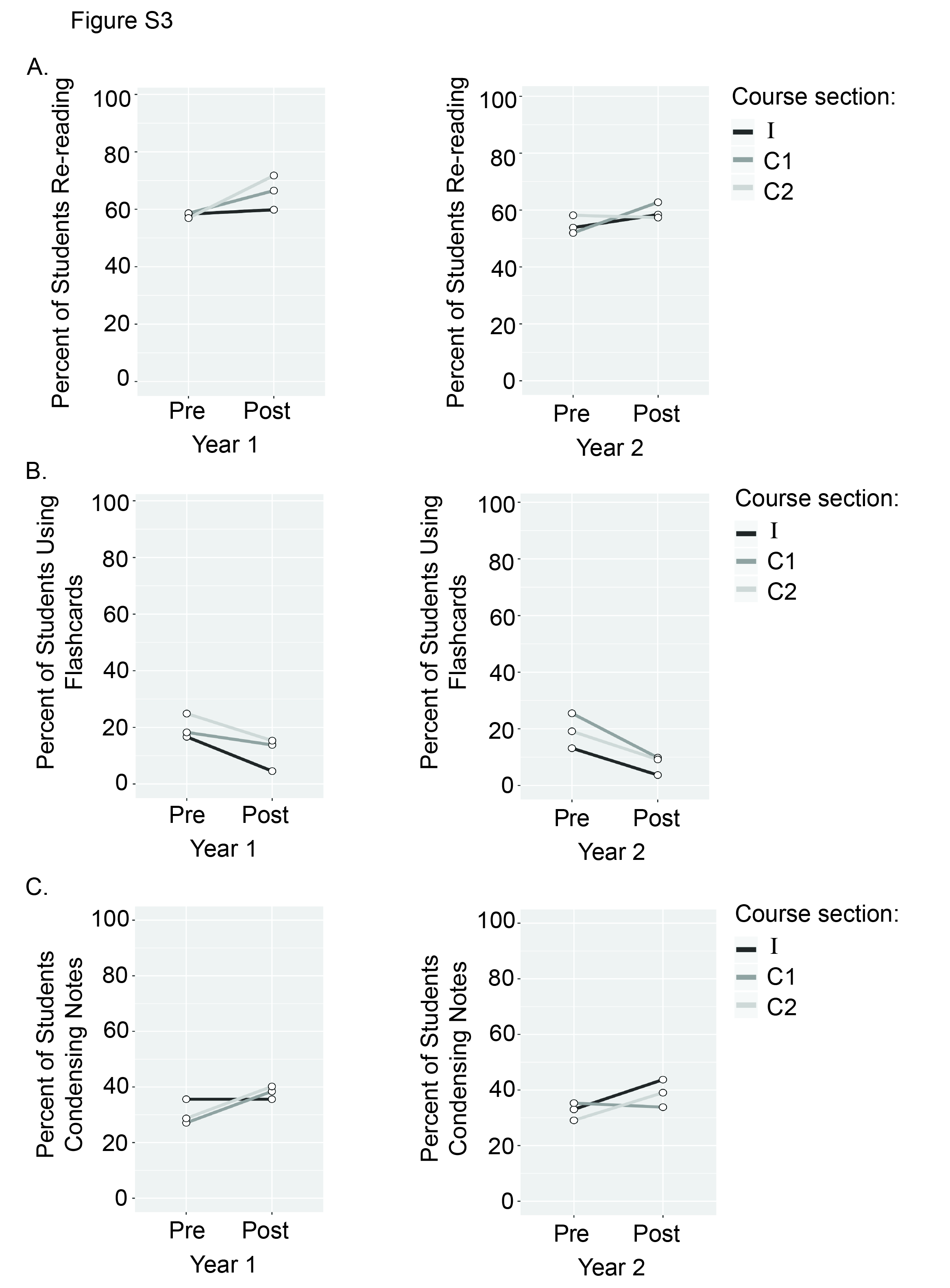

Supplement: S3 Fig — The fraction of student responses that designated either re-reading the textbook (A), using flashcards (B), or condensing notes (C) as one of their top three study strategies on the pre- and post-course survey are reported in the control (C1 and C2) and intervention (I) sections during years 1 and 2 of the study. The study skills intervention did not discuss either of these three study strategies. (TIF) [file pone.0200767.s003.tif]
