# Supplementary material for: How do students study in STEM courses? Findings from a light-touch intervention and its relevance for underrepresented students
Source: PLoS One. 2018 Jul 31;13(7):e0200767. doi: 10.1371/journal.pone.0200767 (PMC6067695; doi:10.1371/journal.pone.0200767)
Supplement: S1 File — (PDF) [file pone.0200767.s004.pdf]

Supporting File 1. Study Skills Intervention Slides

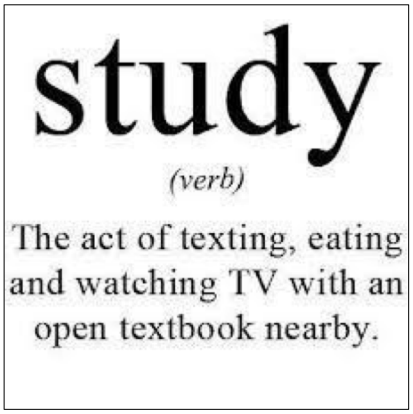

What are you doing?

When are you doing it?

1

What are you doing?

- Discuss with your neighbor:
  - How you have been preparing for the class so far?
  - How do you typically prepare for exams?

2

What are you doing?

- Discuss with your neighbor:
  - How you have been preparing for the class so far?
  - How do you typically prepare for exams?

Rank these studying methods from least useful to most useful in terms of how much you will retain of what you learned

A – reading a textbook

B – answering questions (self-testing)

C – listening to a lecture

D – discussing with your classmates

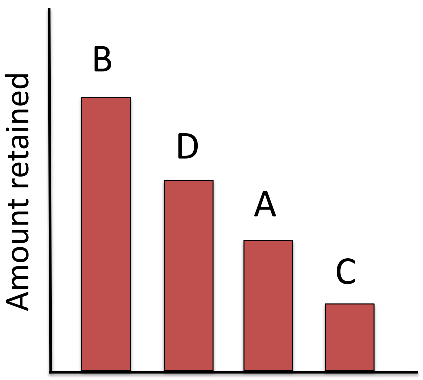

Self-testing is key!

The Learning Pyramid*

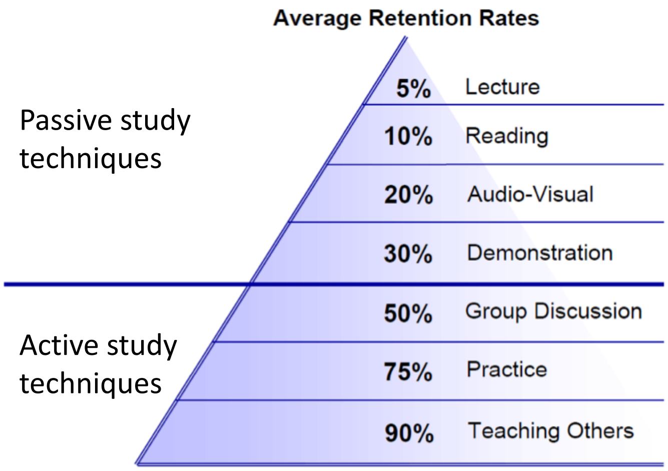

*Adapted from National Training Laboratories. Bethel, Maine

Effective Self-Testing Techniques

- Answering practice questions
- Recalling course concepts from memory
- Explaining course concepts to classmates (or yourself)

- Self-testing reveals what you truly know and what you truly don't

When are you doing it?

- How do you typically prepare for a quiz?

When are you doing it?

2016 APRIL

SUN	MON	TUE	WED	THU	FRI	SAT
					1	2
3	4	5	6<br>HW 1 due	7	8	9
10	11	12<br>STUDY!!	13<br>Quiz 1	14	15	16

When are you doing it?

2016 APRIL

SUN	MON	TUE	WED	THU	FRI	SAT
					1	2
3 Study	4 Study	5 Study	6 HW 1 due	7 Study	8	9
10 Study	11 Study	12 Study	13 Quiz 1	14	15	16

When are you doing it?

- **Schedule** your studying
  - Using google calendar, other apps

Send yourself reminders before exams, HW due dates

Ask yourself:

- Do you utilize self-testing?
- Are you taking an active role in your studying?
- Are your spacing out your studying?

Specific Ways to Practice?

- **In lecture!!!**
- Weekly HW
- Practice Quiz
