# Supplementary material for: How do students study in STEM courses? Findings from a light-touch intervention and its relevance for underrepresented students
Source: PLoS One. 2018 Jul 31;13(7):e0200767. doi: 10.1371/journal.pone.0200767 (PMC6067695; doi:10.1371/journal.pone.0200767)
Supplement: S2 File — (PDF) [file pone.0200767.s005.pdf]

Supporting File 2. Pre- and Post-Survey Study Skills Items

Adapted from Morehead, Rhodes, & DeLozier (2016) and Kornell & Bjork (2007)

Pre-Survey

1. Which of the following best describes your study patterns?
   - I most often space out my study sessions over multiple days/weeks
   - I most often do my studying right before the test

2. Select the top 3 study strategies you use most regularly. Please select ONLY 3.
   - Test yourself with questions or practice problems
   - Use flashcards
   - Reread chapters, articles, notes, etc.
   - Underlining or highlighting while reading
   - Recopy your notes word-for-word
   - Condensing/Summarizing your notes
   - Recopy your notes from memory
   - Make diagrams, charts, or pictures
   - Study with friends
   - Absorbing lots of information the night before the test
   - Watch/listen to recorded lessons either by instructor or from outside source (Khan Academy, Youtube, etc.).
   - Other

3. When studying, how do you generally decide what class to study for first? (open-ended)

Post-Survey

1. Which of the following best describes your study patterns **for this course**?
   - I most often space out my study sessions over multiple days/weeks
   - I most often do my studying right before the test

2. Select the top 3 study strategies you use most regularly **in this course this quarter**. Please select ONLY 3.
   - Test yourself with questions or practice problems
   - Use flashcards
   - Reread chapters, articles, notes, etc.
   - Underlining or highlighting while reading
   - Recopy your notes word-for-word
   - Condensing/Summarizing your notes
   - Recopy your notes from memory
   - Make diagrams, charts, or pictures
   - Study with friends
   - Absorbing lots of information the night before the test
   - Watch/listen to recorded lessons either by instructor or from outside source (Khan Academy, Youtube, etc.).
   - Other

3. When studying, how do you generally decide what class to study for first? (open-ended)
