# Supplementary material for: How do students study in STEM courses? Findings from a light-touch intervention and its relevance for underrepresented students
Source: PLoS One. 2018 Jul 31;13(7):e0200767. doi: 10.1371/journal.pone.0200767 (PMC6067695; doi:10.1371/journal.pone.0200767)
Supplement: S1 Table — Post-hoc tests were used to examine proportion differences for the different spacing and self-testing categories between the intervention and control courses. Maintained use means the student reported using that strategy on both the pre- and post-survey, started using means the student only reported using the strategy on the post, stopped using means the student only reported using the strategy on the pre, and never used means the student did not report using the strategy on either the pre- or post-survey. Note: Ns for each section are the following: Intervention Course = 132, Control Course B = 203, Control Course C = 209. (PDF) [file pone.0200767.s006.pdf]

Table S1. Post-hoc chi-square analysis of spacing and self-testing categories and course section (Year 1).

		Intervention vs. Control Section 1 (n = 335)		Intervention vs. Control Section 2 (n = 341)	
		Raw *p*-value	Adjusted *p*-value	Raw *p*-value	Adjusted *p*-value
Spacing Category Comparisons	Never Used vs. Stopped Using	0.009	0.056	0.071	0.424
	Never Used vs. Started Using	0.538	1.000	0.154	0.922
	Never Used vs. Maintained Use	0.895	1.000	0.017	0.101
	Stopped Using vs. Started Using	0.005	0.032	0.006	0.038
	Stopped Using vs. Maintained Use	0.002	0.014	0.001	0.003
	Started Using vs. Maintained Use	0.567	1.000	1.000	1.000
Self-Testing Category Comparisons	Never Used vs. Stopped Using	1.000	1.000	0.571	1.000
	Never Used vs. Started Using	0.000	0.000	0.001	0.007
	Never Used vs. Maintained Use	0.000	0.001	0.008	0.050
	Stopped Using vs. Started Using	0.000	0.000	0.000	0.001
	Stopped Using vs. Maintained Use	0.000	0.001	0.001	0.004
	Started Using vs. Maintained Use	0.226	1.000	0.183	1.000

Post-hoc tests were used to examine proportion differences for the different spacing and self-testing categories between the intervention and control courses. *Maintained use* means the student reported using that strategy on both the pre- and post-survey, *started using* means the student only reported using the strategy on the post, *stopped using* means the student only reported using the strategy on the pre, and *never used* means the student did not report using the strategy on either the pre- or post-survey. Note: Ns for each section are the following: Intervention Course = 132, Control Course B = 203, Control Course C = 209.
